# Supplementary material for: Patterns of Intron Gain and Loss in Fungi
Source: PLoS Biol. 2004 Nov 30;2(12):e422. doi: 10.1371/journal.pbio.0020422 (PMC532390; doi:10.1371/journal.pbio.0020422)
Supplement: Table S1 — Also available at http://genes.mit.edu/NielsenEtAl/. (4.3 MB ZIP). [file pbio.0020422.st001.zip › NielsenEtAl/html/1266.html]

AN8862.1.NCU01440.1.MG06923.1.FG07469.1


```
 CLUSTAL W (1.82) Multiple Sequence Alignments - Introns Inserted


Sequence 1: NCU01440.1	1568 aa
Sequence 2: MG06923.1	1590 aa
Sequence 3: FG07469.1	1583 aa
Sequence 4: AN8862.1	1569 aa
Alignment Length: 1599 aa
Number Identitical Residues: 1106 aa
Alignment Score (without introns) 46772


MG06923.1 	MAETYDVGTRAWQPDANEGWVASEVVNKKVDGSKVILTFKLENGE0TKEVQVTLEALQSG
NCU01440.1	MSESYDVGTRAWQPDPTEGWVASEVVKKTVDGNKVTLIFECENGE0TKTVEVSLEALQSG
FG07469.1 	MASAYEVGTRAWQPDAAEGWVASELVNKTVDGSKVKLTFQLENGE0TKNIEVSAEALQSG
AN8862.1  	MAHNYEVGTRAWQPDATEGWVASEVKEKLVDGDKVRLVFVLENGE0PKEIETTQAELQVD
          	*:  *:*********. *******: :* ***.** * *  **** .* ::.:   ** .

MG06923.1 	SDPSLPPLMNPTMLEASDDLTNLSHLNEPAV1LQAIKLRYAQKEIYTYSGIVLIATNPFA
NCU01440.1	GHESLPPLMNPTMLEASDDLTNLSHLNEPAV1LQAIRLRYAQKEIYTYSGIVLIATNPFA
FG07469.1 	SDPSLPPLMNPTMLEASDDLTNLSHLNEPAV1LQAIRLRYLQKEIYTYSGIVLIATNPFA
AN8862.1  	NNPNLPPLMNPAMLEASEDLTNLSHLNEPAV1LQAIKLRYAQKEIYTYSGIVLIATNPFA
          	.. .*******:*****:************* ****:*** *******************

MG06923.1 	RVDSLYVPGMVQVYAGKQRATQAPHLFAIAEEAFM~DMLRDNKNQTVVVSGESGAGKTVS
NCU01440.1	RVDSLYVPGMVQVYAGKQRATQAPHLFAIAEEAFM2DMLRDGKNQTIVVSGESGAGKTVS
FG07469.1 	RVDSLYVPGMVQVYAGRQRATQAPHLFAIAEEAFI2DMIRDKKNQTVVVSGESGAGKTVS
AN8862.1  	RVDSLYVPQMVQVYAGKHRASQAPHLFAIAEEAFG2DMLRDGKNQTIVVSGESGAGKTVS
          	******** *******::**:*************  **:** ****:*************

MG06923.1 	AKYIMRYFATRESPNNPGGKSKKG-AEAMSETEEQILATNPIMEAFGNAKTTRNDNSSRF
NCU01440.1	AKYIMRYFATRESPDSPGSRVKKGGHESMSQTEEAILATNPIMEAFGNAKTTRNDNSSRF
FG07469.1 	AKYIMRYFATRESPDNPGGRSKRG-SESMSETEEQILATNPIMEAFGNAKTTRNDNSSRF
AN8862.1  	AKYIMRYFATRESSDQPGKYTTSR-ADAISETEEQILATNPVMEAFGNAKTTRNDNSSRF
          	*************.:.**   .    :::*:*** ******:******************

MG06923.1 	GKYIEIMFDENTNIIGAKIRTYLLERSRLVFQPLKERNYHIFYQLVAGVSDQQRQELGIL
NCU01440.1	GKYIEIMFDKATNIIGAKIRTYLLERSRLVFQPLKERNYHIFYQLVAGVTDKERQELGLL
FG07469.1 	GKYIEIMFDEETNIIGAKIRTYLLERSRLVFQPLKERNYHIFYQLIAGASDQQREELGLL
AN8862.1  	GKYIEIMFDDKTNIVGAKIRTYLLERSRLVFQPLKERNYHIFYQLVAGASDTEKQELGLT
          	*********. ***:******************************:**.:* :::***: 

MG06923.1 	AIEEFEYLNQGNTPTIDGVDDKAEFMATKASLKTIGVTDAQQDEIFKLLAGLLHLGNVKI
NCU01440.1	PVEQFEYLNQGNTPTIDGVDDKAEFNATKASLKTIGVDEGKQTEIFKLLAGLLHLGNVKI
FG07469.1 	PIEEFEYLNQGNCPTIDGVDDKAEFEATQKSLSTIGVTNEQQADIFKLLAGLLHLGNVKI
AN8862.1  	SVEDFDYLNQGGTPIIDGVDDKTEFIATKKSLGTIGVPETIQSEIFRVLAALLHLGNVKI
          	.:*:*:*****. * *******:** **: ** **** :  * :**::**.*********

MG06923.1 	TASRSDSVLAPTEPSLERACAILGIDATEFAKWIVKKQLVTRGEKITSNLSQAQAIVVRD
NCU01440.1	GAMRNDSSLDPSEPSLVKACEILGIDAPEFAKWIVKKQLVTRGEKIVSNLTQAQAIVVRD
FG07469.1 	TASRNDSVLAPNEPSLEKACDILGVKAEEFSRWIVKKQLVTRGEKITSNLSQAQAIVVRD
AN8862.1  	TATRTDSTLSPSEPSLSRACEILGIDANEFAKWIVKKQLITRGEKITSNLTQQQAIVVRD
          	 * *.** * *.**** :** ***:.* **::*******:******.***:* *******

MG06923.1 	SVAKFIYSSLFDWLVDIINRSLATDSVIQRVKCFIGVLDIYGFEHFAKNSFEQFCINYAN
NCU01440.1	SVAKYIYSSLFDWLVEIINHSLASEEVLTRVTSFIGVLDIYGFEHFAKNSFEQFCINYAN
FG07469.1 	SVAKFIYSSMFDWLVDIINTSLASEDVLSRVTSFIGVLDIYGFEHFAKNSFEQFCINYAN
AN8862.1  	SVAKFIYSSLFDWLVDKINRGLATDEVLNKFKSFIGVLDIYGFEHFAKNSFEQFCINYAN
          	****:****:*****: ** .**::.*: :...***************************

MG06923.1 	EKLQQEFNQHVFKLEQEEYLKEQIDWTFIDFADNQPCIDLIEGKLGILSLLDEESRLPMG
NCU01440.1	EKLQQEFNQHVFKLEQEEYLREQIDWTFIDFADNQPCIDLIEGKLGILSLLDEESRLPMG
FG07469.1 	EKLQQEFNQHVFKLEQEEYLREQIDWTFIDFSDNQPCIDLIEGKLGILSLLDEESRLPMG
AN8862.1  	EKLQQEFNQHVFKLEQEEYVREQIDWTFIDFSDNQPCIDLIEAKLGILSLLDEESRLPMG
          	*******************::**********:**********.*****************

MG06923.1 	SDEQFVTKLHHHYSGDKHQFYKKPRFGKSAFTVCHYAVDVTYESEGFIEKNRDTVPDEHM
NCU01440.1	SDEQFVTKLHHNYAADKHKFYKKPRFGKSSFTVCHYAIDVTYESDGFIEKNRDTVPDEHM
FG07469.1 	SDEQFVTKLHHNFTPDKSKFYKKPRFGKSAFTVCHYAIDVTYESEGFIEKNRDTVPDEHM
AN8862.1  	SDEQFVTKLHHNFAADKQKFYKKPRFGKSAFTICHYAVDVTYESDGFIEKNRDTVPDEHM
          	***********::: ** :**********:**:****:******:***************

MG06923.1 	AVLRASSNKFLGQVLDAAASLREKDLASASTAVAKPTAGRKIGVAVNRKPTLGGIFRSSL
NCU01440.1	AVLRASTNQFLVSVLDAASAVREKDLASASSNAVKPAAGRRIGVAVNRKPTLGGIFKSSL
FG07469.1 	AVLRASSNEFLKTVLDAATAVREKDAASSSSNAVKPAAGRKIGVAVNRKPTLGGIFRSSL
AN8862.1  	EILRNSSNNFVKEILDTAASVREKDSAAVSSKPVT-APGRKIGVAINRKPTLGGIFKSSL
          	 :** *:*:*:  :**:*:::**** *: *:  .. :.**:****:**********:***

MG06923.1 	IELMNTINNTDVHYIRCIKPNEAKEAWKFEGPMVLSQLRACGVLETVRISCAGYPTRWTY
NCU01440.1	IELMNTINGTDVHYIRCIKPNEAKEPWKFEGPMVLSQLRACGVLETVRISCAGYPTRWTY
FG07469.1 	IELMSTINNTDVHYIRCIKPNEAKEAWKFEGPMVLSQLRACGVLETVRISCAGYPTRWTY
AN8862.1  	IELMSTINSTDVHYIRCIKPNEAKEAWVFEGPMVLNQLRACGVLETVRISTAGYPTRWTY
          	****.***.****************.* *******.************** *********

MG06923.1 	EEFALRYYMLVPSNQWTAEIRPMANAILTKALGENSGKGTDKYQLGLTKIFFRAGMLAFL
NCU01440.1	EEFALRYYMLVPSTQWTSEIREMANAILTKAFGASTGKGLDKYQLGLTKIFFRAGMLAFL
FG07469.1 	EEFALRYYMLVRSDGWTSEIREMADAILKKALGTSTGKGLDKYQLGLTKIFFRAGMLAFL
AN8862.1  	EEFAIRYYMLCHSSQWTSEIKDMCHAILRKALGDATQQKHDKYQLGLSKIFFRAGMLAFL
          	****:*****  *  **:**: *..*** **:*  : :  *******:************

MG06923.1 	ENLRTSKLNDCAIMIQKNLRARYYR~RRFIAIRESIIRLQATVRGHKARKEAQDLRVTKA
NCU01440.1	ENLRTTRLNDCAILIQKNLKAKYYP1--------------------------QERRTTKA
FG07469.1 	ENLRTTRLNDCAIMIQKNLRAKYYR~QRYLEAREAIILTQSAIRAWKARKSANELRTVKA
AN8862.1  	ENLRTSRLNECAIMIQKNLRCKYYR~RRYLEARLSVLATQSLVRGFLARQRAAEIRRIKA
          	*****::**:***:*****:.:**          :    .:   .  : . : : *  **

MG06923.1 	ATTIQRVWRGHKQRKAFLRIKNDLTLAQAAMKGYLRRKEIMETRVGNAALLIQRVWRSRR
NCU01440.1	AITIQRVWRGYKDRKQFLEVRNDVIRAQAAIKGYMKRKKIMEERVGNAVLIIQRNWRSRQ
FG07469.1 	AITIQRVWRGSKQRKSYLQFRKDMVLFESIAKGYLRRKTILEERLGNAALKIQRSWRSRR
AN8862.1  	ATTIQRVWRGQKERKRYNQIRDNVILLQSLSKGFLCRRNILNSIHGNAAKTIQRAFRSWR
          	* ******** *:** : ..:.::   ::  **:: *: *::   ***.  *** :** :

MG06923.1 	QLRSWRQYRKKIVLIQSLWRGKTARRGYKKVREEARDLKQISYKLENKVVELTQSLGTMK
NCU01440.1	QLRSWRDYRRKVTIVQSLWRGKTARKDYKALRAEARDLKQISYKLENKVVELTQSLGTMK
FG07469.1 	QLKSWRQYRKKVVLIQSLWRGRKARKEYKTIREEARDLKQISYKLENKVVELTQSLGSMK
AN8862.1  	QLRAWRQYRRQVIIVQNLWRGKKARREYKVLREEARDLKQISYKLENKVVELTQYLESLK
          	**::**:**::: ::*.****:.**: ** :* ********************* * ::*

MG06923.1 	TQNKDLKNQVENYENQIKSWKSRHNALELRTKELQTEANQAGIAGAKLEQLEDEYKKLQT
NCU01440.1	NQNRELKTQVENYEGQVAIWRNRHNQLEARAKELQAEANQAGIAAARLEQMEAEMKKLQA
FG07469.1 	ERNKGLASQVENYEGQIKSWKKRHNDLEARTKELQTEANQAGIAVARLQAMEDEMKKLQV
AN8862.1  	RENKSLNSQLENYETQLKSWRSRHNALENRSRELQAEANQAGITAARLTALEEEMSILQQ
          	 .*: * .*:**** *:  *:.*** ** *::***:*******: *:*  :* * . ** 

MG06923.1 	NFDESVANVKRMQQAEAELKDSLRATTTELEAAREDINRSEAEKNNLRQQLVELQDALEI
NCU01440.1	SFEESVANVKRMQEEERELRESLRATSVELDSARIESQRQEAEKNSLRQQLADLQEALEQ
FG07469.1 	AFDESTANIKRMQEEERELRESLRLTNTELETAKRSSTQHEKDNMSLRQELESLRDALEL
AN8862.1  	NHADGQATIKRLQEEERISRDSIRSANQELEKLKQLNAEAESERTSLRQQVIDLEEQLEV
          	 . :. *.:**:*: *   ::*:* :. **:  :    . * :. .***:: .*.: ** 

MG06923.1 	ARRSAPVGN--GEIANGAAPTPSLANGLINLVSAK--KPKRRSAGAEPREMDRYSMAYNP
NCU01440.1	ARRQVPIN---GDILNGNGIAPQVPTGLINLVSSK--KPKRRSAGAEPREMDRYSMAYNP
FG07469.1 	ARRNAPIN---GEIANGTTPGATVPSGLINLVASK--KPKRRSAGAEPRELDRFSGTYNP
AN8862.1  	AKRTLPLQALNGDQQNG-GSVPPPANGLINLVSSKKTKPKRRSAGAERIDTDRFSGAYNP
          	*:*  *:   .*:  **    .  ..******::*.:**********  : **:* :***

MG06923.1 	RPVSMAVTGGGRQ-TLSGSTFVASADTIEMELESLLADEEGLNDEVTVGLIRNLKIPSPN
NCU01440.1	RPVSMAVPGLNRQTTLSGSTFVPGVDSIELELEGLLADEDALNEEVTMGLIRNLKIPSPS
FG07469.1 	RPVSMAVTGTAHRQNLSGSTFLPGVDNIEMELETLLADEDGLNEEVTMGLIRNLKIPSPS
AN8862.1  	RPVSMAIPSALGRQNYSVAALSSGLDSVEAELETLLSEEDELNEEVAIGLIRNLKIPAPN
          	******:..   : . * ::: .. *.:* *** **::*: **:**::*********:*.

MG06923.1 	TNPPPSDKEVLFPSYLINLVTSEMWNNGFVKESERFLANVMQSIQQEVMQHDGEDAINPG
NCU01440.1	TNPPPSDKEVLFPAYLINLVTSEMWNNGFVKESERFLANVMQSIQQEVMQHDGEEAINPG
FG07469.1 	TTPPPSDKEVLFPSYLINLVTSEMWNNGFVKESERFLANVMQSIQQEVMQHDGDEAVNPG
AN8862.1  	STPPPTEKEVLFPAYLINLVTSEMWNNGFVKESERFLANVMQSIQQEVMQHDGDDTISPG
          	:.***::******:***************************************::::.**

MG06923.1 	AFWLSNVHEMLSFVFLAEDWYEAQKTDNYEYDRLLEIVKHDLESLEFNIYHTWMKVLKKK
NCU01440.1	AFWLSNVHEMLSFVFLAEDWYEAQKTDNYEYDRLLEIVKHDLESLEFNIYHTWMKVLKKK
FG07469.1 	AFWLSNVHEMLSFVFLAEDWYEAQKSDNYEYDRLLEIVKHDLESLEFNIYHTWMKVLKKK
AN8862.1  	AFWLSNVHEMLSFVFLAEDWYEAQKTDNYEYDRLLEIVKHDLESLEFNIYHTWMKLLKKK
          	*************************:*****************************:****

MG06923.1 	LHKMIIPAIIESQSLPGFVTNENSRFLGKLLQGNSTPAYSMDNLLSLLNSVFRAMKAYYL
NCU01440.1	LFKMIIPAIIESQSLPGFVTNENNRFLGKLLQSNTAPAYSMDNLLSLLNNVYRAMKAYYL
FG07469.1 	LQKMIIPAIIESQSLPGFVTNESSRFLGKLLQSNSTPAYSMDNLLSLLNSVFRAMKAYYL
AN8862.1  	LYKMIVPAIIESQSLPGFVTNETNRFLGKLLPSNNNPAYSMDNLLSLLNNAYKAMKAFYL
          	* ***:****************..******* .*. *************..::****:**

MG06923.1 	EDSIITQTVTELLRLVGVTAFNDLLMRRNFLSWKRGLQINYNITRIEEWCKSHDMPEGTL
NCU01440.1	EDSIITQTITELLRLVGVTAFNDLLMRRNFLSWKRGLQINYNITRIEEWCKSHDMPEGTL
FG07469.1 	EDSIITQTITELLKLVGVTAFNDLLMRRNFLSWKRGLQINYNITRIEEWCKSHDMPEGTL
AN8862.1  	EDSIITQTVTELLRLVGVTAFNDLLMRRNFLSWKRGLQINYNITRIEEWCKSHDMPEGTL
          	********:****:**********************************************

MG06923.1 	QLEHLM0QATKLLQLKKATLNDIEIIQDICWM2LSPNQIQKLLNQYLVADYEQPINGEIM
NCU01440.1	QLEHLM0QATKLLQLKKATLNDIEIIQDICWM2LSPNQIQKLLNQYLVADYEQPINGEIM
FG07469.1 	QLEHLM0QATKLLQLKKATLNDIEIIQDICWM2LSPNQIQKLLNQYLVADYEQPINGEIM
AN8862.1  	QLEHLM0QATKLLQLKKATLNDIEIIQDICWM2LSPNQIQKLLNQYLVADYEQPINGEIM
          	****** ************************* ***************************

MG06923.1 	KAVASRVTEKSDVLLLQAVDMDDSGPYEIAEPRVITALETYTPSW1LQTPRLKRLAEIVS
NCU01440.1	KAVASRVSEKSDVLLLQAVDMDDSGPYEIAEPRVITALETYTPSW1LQTPRLKRLAEIVS
FG07469.1 	KAVASRVTEKSDVLLLQAVDMDDSGPYEIAEPRVITALETYTPSW1LQTPRLKRLAEIVS
AN8862.1  	KAVASRVTEKSDVLLLTPVDMEDSGPYEIAEPRVITALETYTPSW1LQTPRLKRLAEIVS
          	*******:******** .***:*********************** **************

MG06923.1 	AQAIAQQEKMDMQEYDGEEDEGEDGQQ---QLGQVDEAPEPIGASA
NCU01440.1	QQAIAQQEKMEFGSQAGDYQHGDYDTQSMNDLAEVDEGQEIVEASA
FG07469.1 	AQAIAQQEKLDYTDGE-EYENGHQHELEGVDEVEVEQ---------
AN8862.1  	AQAMAQQDKPENTENG------------------VME---------
          	 **:***:* :  .                    * :
```
